# A traveling SARS-CoV-2 laboratory as part of a pandemic response among vulnerable Brazilian populations

**DOI:** 10.1186/s12889-022-14867-2

**Published:** 2023-01-03

**Authors:** Maria Carolina Elias, Svetoslav Nanev Slavov, Alex Ranieri Jeronimo Lima, Antonio Jorge Martins, Claudia Renata dos Santos Barros, Debora Botequio Moretti, Eduardo L. Araujo, Elaine Cristina Marqueze, Gabriela Ribeiro, Gabriela Mauric Frossard Ribeiro, Jardelina Souza Todao Bernardino, Jaqueline Reginato Koser, Luan Gaspar Clemente, Luiz Aurelio Campos Crispin, Luiz Carlos Junior Alcantara, Luiz Lehmann Coutinho, Marta Giovanetti, Quetura Oliveira Silva, Raul Machado Neto, Ricardo Haddad, Simone Kashima, Vincent Louis Viala, Dimas Tadeu Covas, Sandra Coccuzzo Sampaio

**Affiliations:** 1 Instituto Butantan, São Paulo, Brazil; 2 Center of Toxins, Immune Response and Cell Signaling – CeTICSInstituto Butantan, São Paulo, 05503-900 Brazil; 3 University of São Paulo, Ribeirão Preto Medical School, Blood Center of Ribeirão Preto, Ribeirão Preto, Brazil; 4 Loccus, São Paulo, Brazil; 5 Centro de Genomica Funcional, ESALQ-USP, Piracicaba, São Paulo Brasil; 6 Instituto René Rachou, Fundação Oswaldo Cruz, Belo Horizonte, Minas Gerais Brazil

**Keywords:** Brazil, COVID-19, SARS-CoV-2, LabMovel, Real-time detection, Molecular surveillance

## Abstract

**Background:**

Brazil has been dramatically hit by the SARS-CoV-2 pandemic and is a world leader in COVID-19 morbidity and mortality. Additionally, the largest country of Latin America has been a continuous source of SARS-CoV-2 variants and shows extraordinary variability of the pandemic strains probably related to the country´s outstanding position as a Latin American economical and transportation hub. Not all regions of the country show sufficient infrastructure for SARS-CoV-2 diagnosis and genotyping which can negatively impact the pandemic response.

**Methods:**

Due to this reason and to disburden the diagnostic system of the inner São Paulo State, the Butantan Institute established the Mobile Laboratory (in Portuguese: LabMovel) for SARS-CoV-2 testing which started a trip of the most important “hotspots” of the most populous Brazilian region. The LabMovel initiated in two important cities of the State: Aparecida do Norte (an important religious center) and the Baixada Santista region which incorporates the port of Santos, the busiest in Latin America. The LabMovel was fully equipped with an automatized system for SARS-CoV-2 diagnosis and sequencing/genotyping. It also integrated the laboratory systems for patient records and results divulgation including in the Federal Brazilian Healthcare System.

**Results:**

Currently,16,678 samples were tested, among them 1,217 from Aparecida and 4,564 from Baixada Santista. We tracked the delta introductio in the tested regions with its high diversification. The established mobile SARS-CoV-2 laboratory had a major impact on the Public Health System of the included cities including timely delivery of the results to the healthcare agents and the Federal Healthcare system, evaluation of the vaccination status of the positive individuals in the background of exponential vaccination process in Brazil and scientific and technological divulgation of the fieldwork to the most vulnerable populations.

**Conclusions:**

The SARS-CoV-2 pandemic has demonstrated worldwide the importance of science to fight against this viral agent and the LabMovel shows that it is possible to integrate researchers, clinicians, healthcare workers and patients to take rapid actions that can in fact mitigate this and other epidemiological situations.

**Supplementary Information:**

The online version contains supplementary material available at 10.1186/s12889-022-14867-2.

## Background

Brazil has been dramatically affected by the COVID-19 pandemic, resulting in some of the highest rates of COVID-19 morbidity and mortality worldwide. From the confirmation of the first SARS-CoV-2 infection case in Brazil in February 2020 in the city of São Paulo [1], São Paulo State has been considered a national epicenter of the pandemic, showing the highest indexes of COVID-19 incidence, morbidity, and mortality (Brazilian Ministry Health, 2021). The city is the largest national and international hub with the most important international airports and bus stations in Brazil and South America and is the economic and industrial heart of the country. The state is the most populous and highly industrialized area in Brazil, with the largest and busiest port complex in Latin America, the port of Santos. These specific geographic and economic characteristics of the state have shaped the COVID-19 pandemic in Brazil, contributing not only to the highest SARS-CoV-2 infection indexes in Brazil but also to the extensive circulation of variants of concern (VOCs) and interest (VOIs), many of which have been uniquely described in the state [2]. The Brazilian COVID-19 pandemic has also been a continuous source of new VOCs and, more specifically, the gamma VOC (P.1), which emerged in the Brazilian state of Amazonas (North Brazil) during late 2020 but rapidly spread throughout the whole country [3] and was responsible for the second pandemic wave, which also affected São Paulo State in terms of contingency and diagnostic policies. Currently, this scenario has been shaped by the delta VOC, which was the dominant lineage in the state and is now being replaced by the omicron VOC (https://butantan.gov.br/covid/boletim).

During this alarming scenario at the beginning of 2020, Instituto Butantan, located in the city of São Paulo, was in charge of coordinating the molecular diagnosis for the state territory. A network of 27 public laboratories (mostly from universities) was established, which received daily nasopharyngeal swabs (NPSs) from the 17 Regional Health Departments of the State for routine SARS-CoV-2 diagnosis. The functioning of this network relies on a complex logistic organization providing diagnostic reagents and clinical samples from hospitals and institutions throughout the state and delivering results an average of four days after sample collection. At the beginning of 2021, when SARS-CoV-2 variants were threatening public health, the Butantan Network for Pandemic Alert of SARS-CoV-2 Variants was established to trace VOC and VOI dissemination in São Paulo State. The Butantan network provides rapid information about SARS-CoV-2 variants circulating in the 17 Regional Health Divisions of the State composed of the regions of Metropolitan São Paulo, Baixada Santista, Registro, Taubate, Sorocaba, Campinas, São João da Boa Vista, Piracicaba, Bauru, Ribeirão Preto, Araraquara, Marilia, Araçatuba, Presidente Prudente, São José do Rio Preto, Barretos, and Franca. This initiative allowed us to fully characterize the second gamma wave in the state, circulating VOCs such as the beta variant (B1.351) [2], and the introduction of the delta [4] and omicron VOCs (https://butantan.gov.br/covid/boletim), among others. Although the highest SARSCoV-2 diagnostic potential is attributed to the city of São Paulo, this could not be extrapolated to the whole state territory, which represents an uneven distribution of SARS-CoV-2 detection capabilities, such as time to diagnosis and a timely manner of obtaining SARS-CoV-2 VOC/VOI information. Moreover, after 20 months of the pandemic in Brazil, it is clear that efforts from scientists, researchers, clinicians, and healthcare workers need to be integrated for a rapid and efficient response to the actual and ever-changing challenges imposed by the COVID-19 pandemic.

## Methods

### Sample collection, transportation, labeling

Between August 5, 2021, and September 16, 2021, NPS samples were collected from symptomatic patients suspected of COVID-19 in the Basic Health Units of each municipality. After collection, all samples were sent to LabMovel for SARS-CoV-2 diagnosis and genome sequencing. Briefly, NPS samples were collected in 15 mL Falcon tubes. Tubes were labeled individually with a number that was used in the whole process for sample tracking. They were then transported in refrigerated boxes to LabMovel. Upon receipt, tubes were visually inspected to meet the processing requirements (tube type, proper tag position, undisrupted barcode).

### Nucleic acid extraction and SARS-CoV-2-RT–PCR

SARS-CoV-2 RNA was isolated from 100 µL of liquid in which the NPS was immersed. Samples were delivered in swabs immersed in 3 mL of phosphate-saline buffer solution (PBS).

Sample inactivation was performed using a hermetic automated tool for sample isolation and handling, such as airflow and HEPA filters that works as biosafety level 2. This automation was especially conceived to enforce the lab biosafety capabilities. RNA extraction was then carried out automatically using an Extracta Kit (Loccus) following the manufacturer's instructions. RT–PCR was carried out automatically using a liquid handler (Extracta Prep, Loccus). SARS-CoV-2 RNA detection was performed using a Gene FinderTM COVID-19 Fast RealAmp Kit (OSang Healthcare Co., Ltd.) targeting the viral RdRp, E, and N genes. Amplification was performed in a QuantStudio 5 (Thermo Fisher Scientific). Positive samples with a cycle threshold (Ct) for at least 2 viral genes of Ct < 30 and a positive RNAse P test were submitted to viral genotyping.

### Genomic library preparation and SARS-CoV-2 next-generation sequencing

All positive samples with Ct values < 30 were submitted to SARS-CoV-2 variant identification. Genomic libraries were prepared using COVIDseq (Illumina) in an automated SP-960 system (Loccus/MGI). To evaluate the input concentration of the generated libraries, they were quantified using the FluorQuant system with a FluorQuant High Sensitivity Detection Kit (Loccus). Pooled libraries were sequenced using iSeq (Illumina).

### Decontamination of LabMovel and discard of the biohazardous waste

As no manual sample handling (tube opening, pipetting, etc.) was performed outside the equipment, our main concern was related to always maintain all analogic or automated equipment decontaminated. Considering that our stay in each city had a timespan of 3 weeks, we established two types of decontamination protocols: decontamination on arrival and daily decontamination protocol. The first one contemplates a general decontamination. As the container is transported between the cities, initially non-specific cleaning was performed on the whole LabMovel composition using common disinfectant reagents. After this, the laboratory contaminants were eliminated by daily protocols contemplating use of isopropyl alcohol applied on all surfaces followed by application of RNaseZAP™ (Sigma Aldrich) and UV light in between the testing of each sample plate. To certify decontamination efficiency, after these protocols we also run blank plates or known positive/negative SARS-CoV-2 samples to control the whole process from extraction to sequencing.The Daily protocol was performed twice a day, at the beginning and ending of the shift. Partnership with cities government was established and a team of trained professionals collected the waste every day, transporting it to the correct disposal sites according to legislation.

### Sequence assembly

The raw sequence data obtained were submitted to quality control analysis using FastQC [5] software version 0.11.8. Trimming was performed using Trimmomatic [6] version 0.3.9 to select the sequences with the best quality. Only sequences with quality scores > 30 were used. We mapped the trimmed sequences against the SARS-CoV-2 reference (GenBank RefSeq NC_045512.2) using BWA [7] software and SAMtools [8] for read indexing. The mapped files were submitted to refinement using Pilon [9] to obtain the correct indels and insertions. The trimmed sequences were subjected to remapping against the genome refined by Pilon. Finally, we used bcftools [10] for variant calling and seqtk [11] for the assembly of the consensus SARS-CoV-2 genomes. Positions covered by fewer than 10 reads (DP < 10) and bases of quality lower than 30 were considered gaps in coverage and converted to Ns. Coverage values for each genome were calculated using SAMtools version 1.12. We assessed the consensus genome sequence quality using Nextclade version 0.8.1 (https://clades.nextstrain.org). Furthermore, only sequences with more than 20,000 bases representing > 10 × depth were selected for analysis.

### Phylogenetic analysis

For the phylogenetic analysis, we sequenced 79.03% of all samples positive for SARSCoV-2. After assembly and quality assessment, 820 sequences from LabMovel were used. Complete SARS-CoV-2 genome sequences from delta and gamma strains were downloaded from the GISAID database, including sequences with collection dates from July 26, 2021, to September 30, 2021. In this way, 2,862 sequences from Brazil were obtained, and 38,047 sequences were obtained from the global database to perform subsampling. Brazilian sequences were grouped by state, except those from São Paulo, and 7% from each state was retrieved; sequences from São Paulo State were grouped by city, and 3% of each city was recovered; this resulted in a Brazilian background dataset of 360 sequences. Global samples were grouped by continent, and 0.7% from each continent was retrieved, resulting in 258 sequences. Additionally, one alpha sample and the Wuhan-Hu-1 sequences were added to the dataset, resulting in a total of 1,440 sequences (Table S1).

For phylogenetic analysis, sequence alignment was performed using MAFFT version 7.475 [12] and the alignment was manually curated to remove artifacts using Aliview [13]. Maximum likelihood (ML) phylogenetic trees were estimated using FastTree MP version 2.1.11, applying the ML algorithm with statistical support of bootstrapping with 1000 replicates.

### Statistical analysis

Poisson regression with robust variance was performed to identify factors associated with positive results for COVID-19. The dependent variable was the positive result for COVID-19, and the independent variables were vaccine schedule, age, sex, comorbidities, and presence of symptoms at the time of the test. Independent variables with *p* < 0.20 in the bivariate analyses were tested in the multivariate model, in decreasing order of statistical significance (stepwise forward technique). In the final model, the significance level was 5%. We used Stata 14.0 (Stata Corp., Texas, USA) for statistical analyses.

## Results and discussion

### Establishing a traveling laboratory for SARS-CoV-2 diagnosis and sequencing: the LabMovel

**Fig. 1:**
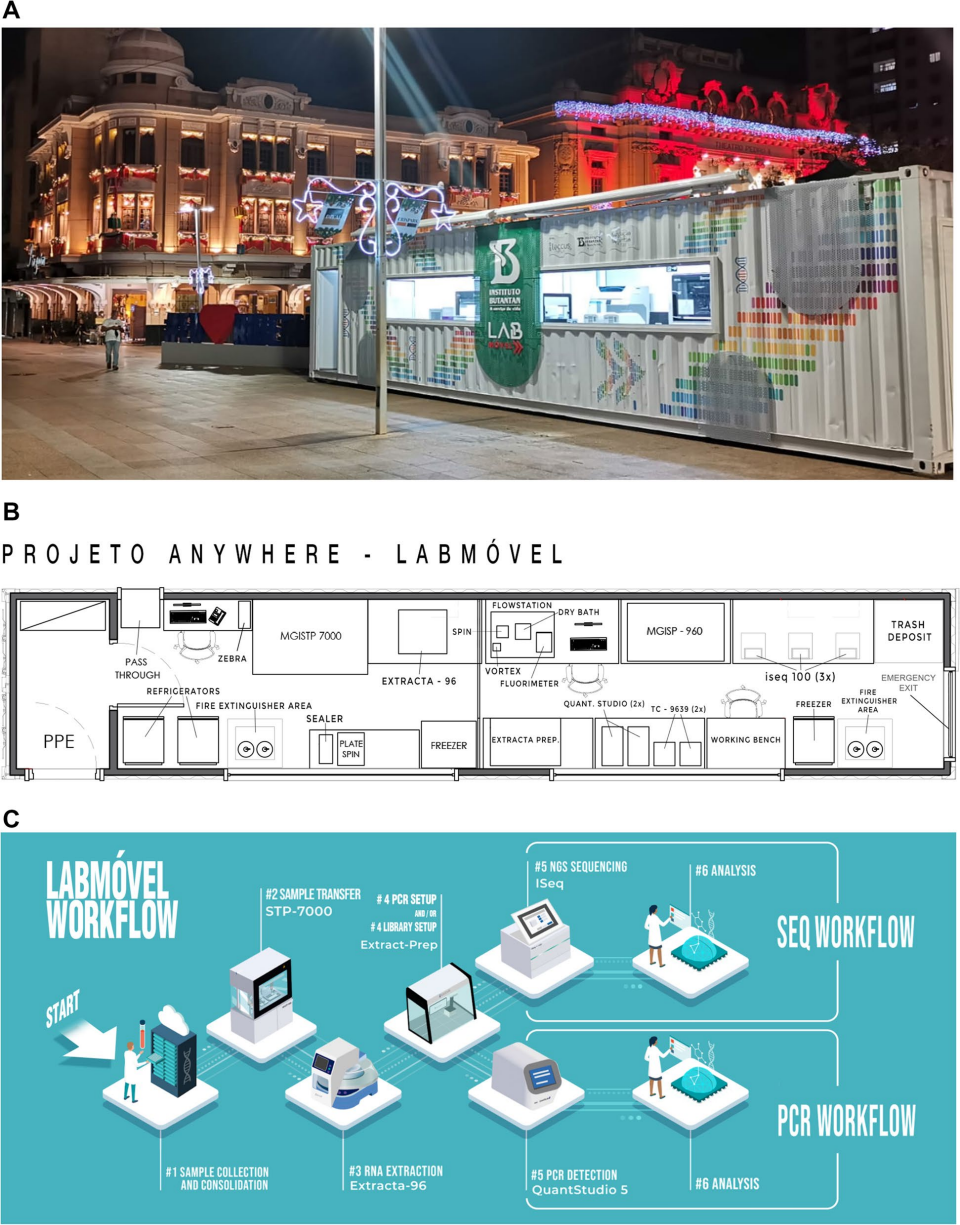
LabMovel and its organization. **A**) Picture of LabMovel at Ribeirão Preto, an inner São Paulo State city. **B**) Internal organization of LabMovel, showing the pipetting robot system that allows the manipulation of samples at LabMovel (MGISTP7000), the pipetting robot that performs RNA extraction (Extracta-96) and PCR preparation (MGISP-960), RT-PCRs machines (Quat-studio), and PCR (TC-9639) and sequencing (iSeq 100) machines. **c**. Workflow of diagnosis and sequencing of SARS-CoV-2. Figure of own authorship

In response to the importance of reducing the interval between sample collection and test result and the need to unify scientists and healthcare agents as well as provide effective communication regarding the importance of science for the general population, we idealized a traveling laboratory (Fig. 1) that could reach (i) cities with a shortage of diagnostic tests; (ii) cities with a high influx of people; and (iii) cities with a lack of sample delivery logistics. This traveling laboratory was named in Portuguese LabMovel (Mobile Lab) and was a result of a partnership between a public institution (Instituto Butantan) and a private company (Loccus). The equipment was provided by Loccus and Illumina. The consumables were funded by Loccus, the Butantan Foundation and FAPESP. All of the diagnostic and sequencing testes were free of charge for the general population.

The LabMovel laboratory infrastructure could be easily visualized from outside (glass windows), and it has an analysis capacity of 7000 samples/day for viral diagnosis and generation of 288 complete SARS-CoV-2 genomes per week. It was equipped with infrastructure allowing a complete workflow from NPS arrival to SARS-CoV-2 diagnosis, next-generation sequencing (NGS) library preparation, SARS-CoV-2 sequencing and variant tracking. The sample processing, SARS-CoV-2 diagnosis and sequencing were performed by four technicians who received training for key processes like extraction, qPCR and sequencing*.* Additionally, we established the Tayna system for patient registration, which consists of a remote laboratory management system allowing registration, collection of clinical data, and delivery of test results. In the first two cities visited by LabMovel, the diagnostic results were delivered in an interval of 24 h by this system directly to the individual's cell phone/email or health unit, significantly reducing the risk of circulation of positive SARS-CoV-2 individuals. Additionally, the median time between sample collection and SARS-CoV-2 variant identification was 8 days due to the necessity of the accumulation of enough samples to complete the NGS plate.

### LabMovel route and diagnostic and sequencing actions

Initially, LabMovel was positioned in two cities of São Paulo State: i) Aparecida do Norte (also receiving samples from the adjacent cities Guaratingueta, Potim, and Roseira) and ii) the city of Santos (also receiving clinical samples from 7 adjacent cities that compose the Baixada Santista region: Guarujá, Itanhaém, Mongaguá, Peruíbe, Praia Grande, São Vicente, and Cubatão). Aparecida do Norte city was chosen since this region is located on the principal highway that links the cities of São Paulo and Rio de Janeiro, and by this period, Rio de Janeiro was the epicenter of the delta VOC outbreak in Brazil. Additionally, Aparecida do Norte city is the principal religious and pilgrimage center in Brazil and experiences a significant human influx. The largest and busiest port in South America, the port of Santos, is located in the city of Santos. During the stay of LabMovel in the city of Aparecida do Norte (between the 5th and 26th of August 2021), the median SARS-CoV-2 incidence (number of positives/total number of tests applied) in the region was 33.9%. By this period, the vaccine coverage with only one dose was 69.1%, and 27.7% of the tested individuals were completely vaccinated (54.40% received Coronavac and 40.22% received AstraZeneca). The median SARS-CoV-2 incidence in the region of Santos city between August 27th, 2021, and September 16th, 2021, was 13.8%. The incomplete vaccine coverage was 74.4% (only one vaccination dose), and 41.1% of the tested individuals had received a complete vaccination schedule (48.47% of them received Coronavac and 39.02% received AstraZeneca). Positioned at Aparecida do Norte, LabMovel performed 1217 tests, and 351 genomes were sequenced (84.1% of the positive samples), while in Santos, LabMovel performed 4564 tests, and 463 genomes were sequenced (75.28% of the positive samples).

**Fig. 2:**
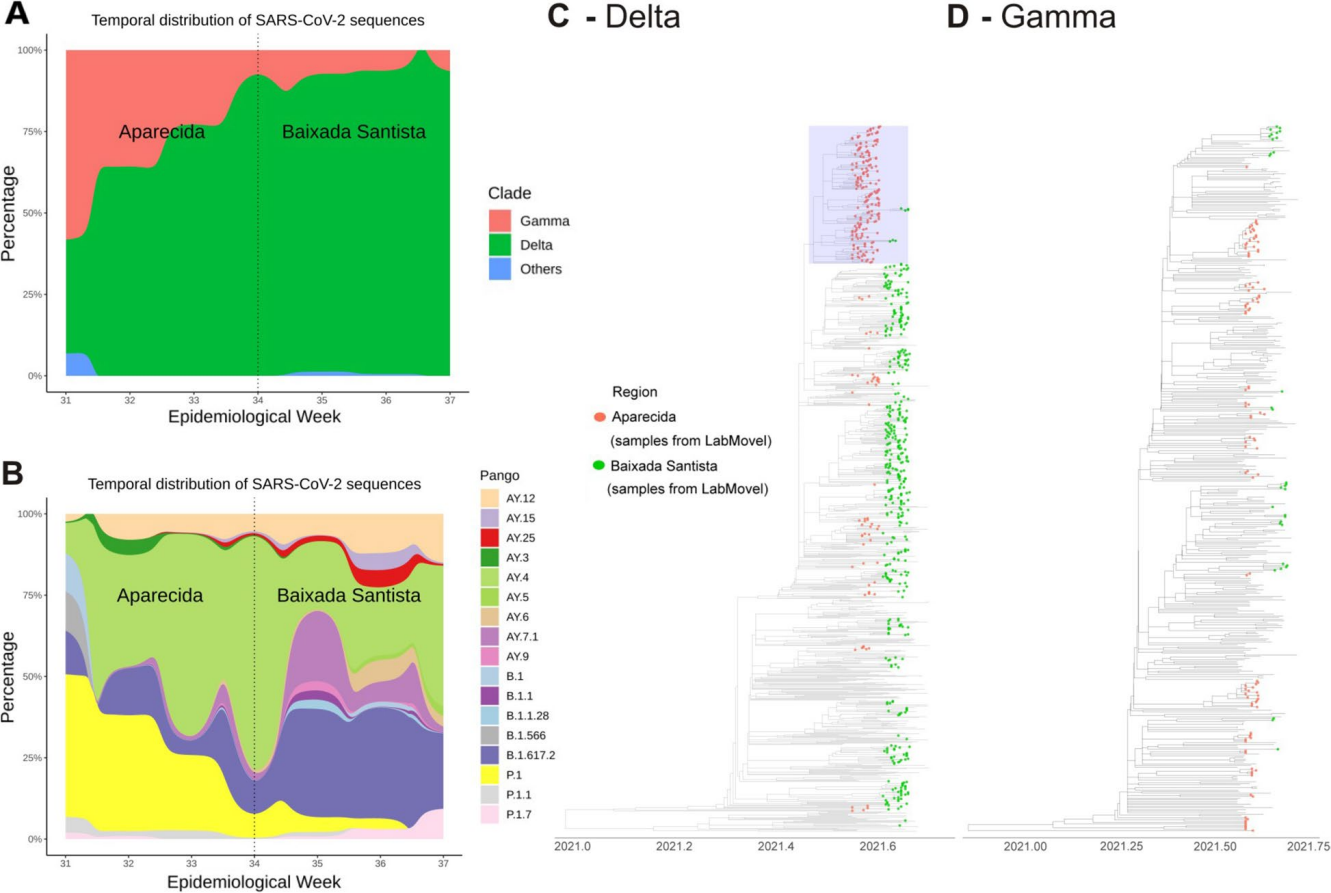
Genomic surveillance of SARS-CoV-2 variants by LabMovel. **A**-**B**. Frequency of SARS-CoV-2 variants in the tested population according to the position of LabMovel (for information about the prevalence of positive cases, see Fig. 3). **C**-**D**. Genomic characterization of SARS-CoV-2 variants of concern (VOCs; delta-gamma) circulating in the location where LabMovel was positioned. Time-stamped maximum likelihood (ML) phylogenetic tree, including the 820 newly sequenced isolates obtained in this study as well as a representative dataset obtained by subsampling containing 360 Brazilian gamma and delta strains and 258 worldwide obtained from GISAID (https://www.gisaid.org) as of September 2021. The sequences obtained in this study are presented as green (Baixada Santista) and red (Aparecida) dots. The delta VOCs obtained from the Baixada Santista region are randomly interspersed within the phylogenetic tree, while the ones from Aparecida do Norte form a monophyletic cluster (see the box)

The performed phylogenetic analysis was consistent with the dissemination of two SARS-CoV-2 VOCs, the gamma and delta VOCs. The period when molecular surveillance was performed coincided with the gradual replacement of the gamma VOC with the delta VOC, which can be observed in Fig. 2A and B. The phylogenetic analysis of the delta (Fig. 2C) and gamma (Fig. 2D) VOCs revealed that both of them were randomly interspersed with other reference strains obtained from Brazil and worldwide, highlighting that multiple independent introduction events occurred over time. However, we observed a large monophyletic cluster composed of AY.4 strains, 75.5% of which were obtained from the city of Aparecida do Norte, which may be related to the sustained transmission of the AY.4 sublineage in this region. Moreover, 59.7% of all delta VOC sequences obtained from the region of Aparecida do Norte belonged to AY.4. In contrast, the delta VOCs obtained from the Baixada Santista region were highly diverse and interspersed with other delta strains obtained from Brazil and worldwide, which can be related to multiple introductions and reintroductions, probably due to the presence of the largest port in the country. Considering all the positive samples, only 16.4% of the total number of strains was classified as gamma. This can be related to the substitution of the gamma VOC by the delta VOC in these Brazilian regions (Fig. 2A and B). Therefore, LabMovel was responsible for providing information on the genetic diversity of variants in the tested regions, revealing the gradual substitution of the gamma VOC by the delta VOC (especially lineages B.1.617.2 and AY.4), which can be observed in the dispersion plot (Fig. 2A and B). The obtained sequencing results show the importance of molecular surveillance for the detection of novel SARS-CoV-2 variants that helps elucidate SARS-CoV-2 dynamics and dispersion [14].

It is challenging for Brazilian scientists to link the metadata of patients with detected SARSCoV-2 variants. For this reason, we established a questionnaire that was completed by each patient tested in LabMovel. This information permitted us to link the positive SARS-CoV-2 results with the vaccination status of the patients. To validate this approach, we used bivariate and multivariate models to analyze the factors associated with SARS-CoV-2 positivity. Using a bivariate model, we observed that incomplete and complete vaccination protected against COVID-19 and was related to negative diagnostic results. Moreover, male individuals had a greater risk than female individuals for positive SARS-CoV-2 PCR results (Table 1). The same result was obtained with the multivariate model (Table 1). The observed findings were similar to those of an American study that observed a reduced attack rate in completely vaccinated individuals, especially elderly individuals [15], and to those of studies [16] that demonstrated a higher susceptibility to SARS-CoV-2 in individuals above 60 years of age.

**Table 1:** Crude and adjusted prevalence ratio of factors associated with positive results for COVID-19

	Bivariate		Multivariate	
	PR_crude_	(95% CI)	PR_adj_	(95% CI)
**Vaccination**				
No vaccination	1		1	
Incomplete vaccination	0.86	(0.76; 0.98)	0.86	(0.75; 0.98)
Complete vaccination	0.66	(0.57; 0.76)	0.63	(0.54; 0.73)
**Age**				
< 24 years	1		1	
25 to 59 years	1.01	(0.88; 1.16)	1.14	(0.99; 1.31)
60 years or more	1.09	(0.91; 1.31)	1.82	(1.11; 1.62)
**Sex**				
Female	1		1	
Male	1.25	(1.12; 1.40)	1.23	(1.09; 1.37)

*Abbreviations, PR* Prevalence ratio

## Conclusions

**Fig. 3:**
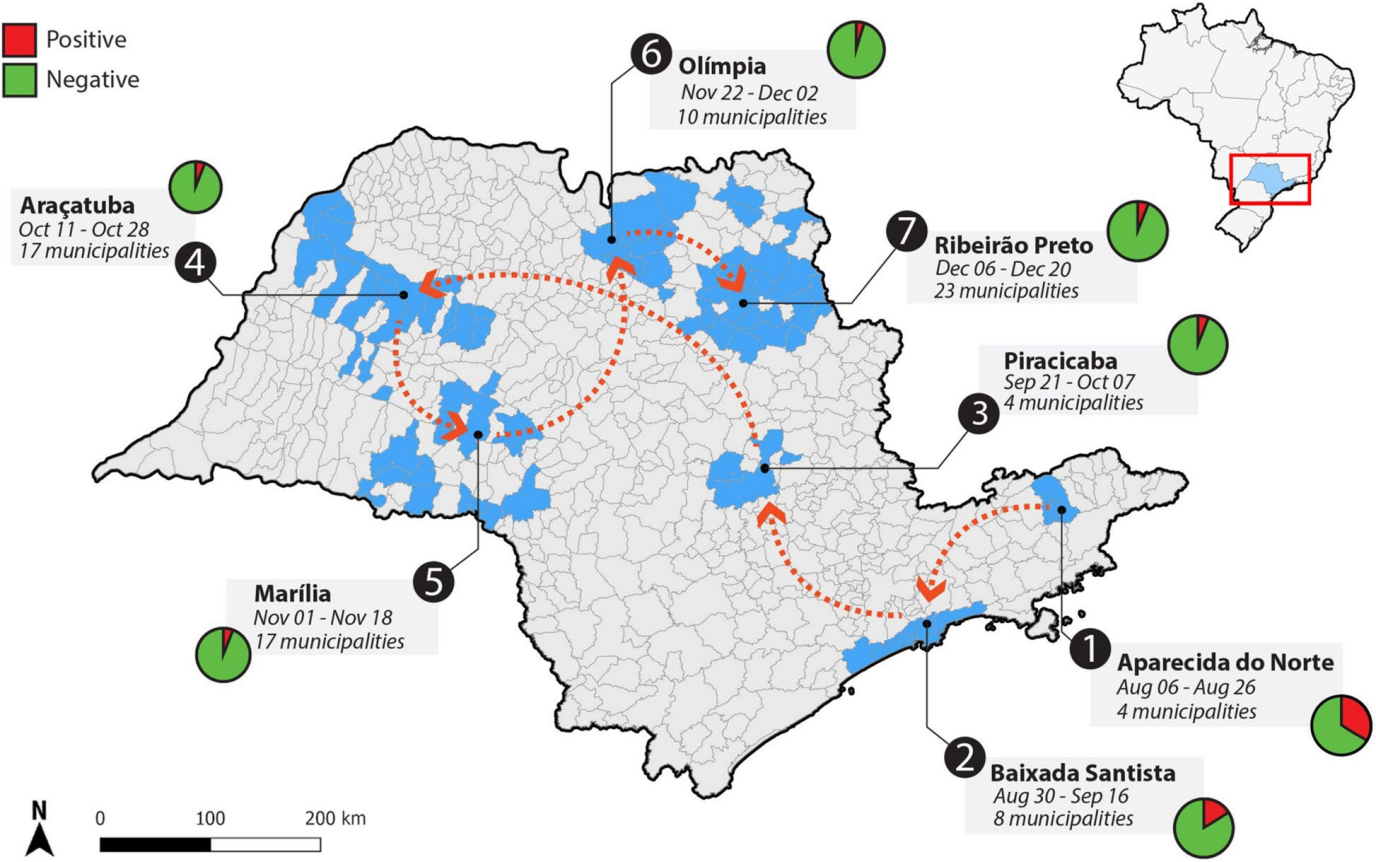
Current itinerary of LabMovel in São Paulo State, Brazil. The map on the right side shows the position of São Paulo State in Brazil. On the left side, the map shows the itinerary of LabMovel across the territory of São Paulo State (red line). Blue regions show municipalities that were attended by LabMovel with the respective prevalence of SARS-CoV2 infection in the tested population. The size of circles is related to the number of tests. The map was plotted using GeoPandas (v 0.10.2), data from IBGE (Instituto Brasileiro de Geografia e Estatística https://geoftp.ibge.gov.br/organizacao_do_territorio/malhas_territoriais/malhas_municipais/ municipio_2021/UFs/SP/SP_Municipios_2021.zip) and edited using Adobe Photoshop Version 14.1.2 (https://www.adobe.com/)

In conclusion, the most important advantages offered by LabMovel were the following: (i) timely delivery of the results that used to be 5–7 days, was was achieved considering the period between sample collection and the result report. The SARS-CoV-2 diagnosis results obtained within 24 h allowed workers to stay away for a minimum possible time (in the case of a negative result) and facilitated rapid contact tracing; (ii) the testing of SARS-CoV-2 in the study locations by the LabMovel increased with 25% compared to one week before the arrival of Labmovel; (iii) the LabMovel software system delivered the diagnostic data directly to the Federal Healthcare System (SUS, Sistema Único da Saúde), which exempts the public health agents from the submission of these results (20–30 min per positive case); (iv) Real-time information of the circulating SARS-CoV-2 variants was provided; (v) Implementation of the questionnaire allowed the relation of metadata with the diagnostic and sequencing results; (vi) Easy visualization of LabMovel allowed the population to be familiarized with the diagnostic and sequencing procedures, which had a scientific and educational impact. Scientific concepts such as “genome”, “PCR”, “mutation” and “variant” were largely explained to the most vulnerable Brazilian population. Due to the importance of the actions of LabMovel, it is currently being continued. LabMovel disrupted paradigms in public health care, showing the importance of the collaboration made up of scientists, health professionals, logistics, and government bodies responsible for public policy measures. Figure 3 shows the itinerary that LabMovel covered until December 2021, reaching the regions of Piracicaba, Araçatuba, Marilia, Olímpia, and Ribeirão Preto and traveling from the starting point (Aparecida Norte) to the most recent destination (Ribeirão Preto) for a total distance of ~ 1,573 km in 135 days. In the near future, we will expand the activities of LabMovel by extending the real-time surveillance for important viral agents, such as influenza A and B, dengue, Zika, and yellow fever viruses, in São Paulo State and probably throughout Brazil.

## Supplementary Information

**Additional file 1**.

## Data Availability

All of the sequences generated in this study were deposited in GeneBank and GISAID are publicly available. Their accession numbers are presented in Supplement Table I (Table S1).

## Abbreviations

NPS: Nasopharyngeal swabs
Ct: Cycle threshold
ML: Maximum likelihood
NGS: Next-generation sequencing
